# Science Teachers’ Perceptions and Cognitive Structures About Skill-Based Questions †

**DOI:** 10.3390/bs15101356

**Published:** 2025-10-04

**Authors:** Nail İlhan, Sultan Şan

**Affiliations:** 1 Faculty of Education, Inonu University, Battalgazi 44280, Türkiye; nail.ilhan@inonu.edu.tr; 2 Graduate School of Education, Inonu University, Battalgazi 44000, Türkiye

**Keywords:** cognitive structures, educational assessment, skill-based questions, teacher perceptions, word association test

## Abstract

The study investigates how Turkish science teachers view and understand skill-based questions (SBQs). SBQs aim to assess higher-order cognitive skills, such as critical thinking and problem-solving, in line with global standards like PISA and TIMSS. The data collected via the Word Association Test (WAT) revealed teachers’ conceptual frameworks and attitudes toward SBQs. The most frequently associated terms were ‘cognitive based’ and ‘culturally context consistency’. Teachers expressed mixed perceptions, with concerns about SBQs’ alignment with international standards, literacy, validity, and the stress they impose on students. Statistical analyses showed that teachers’ cognitive structures lack integration, indicating confusion and limited understanding. The findings highlight the need for enhanced teacher training, clearer guidelines and addressing the gaps between policy and practice. This study contributes to educational assessment reforms by emphasizing the importance of supporting teachers in using SBQs effectively.

## 1. Introduction

The prevailing emphasis in educational research on quantifiable and demonstrable alterations gives rise to an effect–response orientation. When education is regarded as a process, with certain independent variables functioning as inputs and the dependent variable acting as the resultant output, the outcome is often a reductionist approach that is likely to produce superficial results. However, it is imperative to emphasize the significance of examining how inputs and outputs are constituted, that is, the manner in which stakeholders interpret occurrences through the lens of their specific processes, and to comprehend the mechanisms by which they construct knowledge. Skill-based questions (SBQ) have been developed for the purpose of assessing higher-order cognitive abilities in modern education systems. The purpose of these questions is to assess students’ capacity to apply knowledge in real-world contexts (MoNE, 2019). Nevertheless, effective implementation of SBQ is predicated upon a comprehensive understanding of how educators interpret and conceptually construe such questions.

SBQs are questions designed in the context of daily life, in accordance with the curriculum outcomes, and assess high-level cognitive skills (Şan & İlhan, 2022). These questions emphasize skills such as critical thinking, creative thinking and problem solving (see Agustina et al., 2021; Al Abadi & Salem, 2018). The measurement of students’ cognitive skills in relation to science concepts has been investigated since kindergarten (İlhan & Tosun, 2016). Within the Turkish education system, the science curriculum encompasses domain-specific competencies, including science process skills, life skills, and engineering and design skills (MoNE, 2018). As MoNE (2018) state, high school entrance exams assess a range of skills including reading comprehension, interpretation, inference, problem-solving, analysis, critical thinking and scientific process skills. In terms of global assessment, the Programme for International Student Assessment (PISA) and the Trends in International Mathematics and Science Study (TIMSS) are two prominent evaluations which utilize SBQ to evaluate students’ scientific literacy and application skills (see MoNE, 2020; MoNE, 2019 for further details). To illustrate this point, a 2018 investigation into the cognitive levels of science questions within PISA 2018 emphasized the diversity and intricacy of such questions (Syahrial et al., 2022). In addition, a study of eighth-grade students’ proficiency in PISA mathematics problem-solving revealed the importance of effective teaching strategies (Wulandari & Jailani, 2018).

In the course of implementing the SBQ, a number of challenges were identified. A body of research indicates that such questions have the potential to induce stress and anxiety in students preparing for examinations (see Bayburtlu, 2021; Tortop et al., 2022). Concerns regarding the compatibility of SBQ with existing textbooks have been expressed by educators (Erden, 2020). Furthermore, a divergence of opinion exists among educators with respect to the calibre and sufficiency of the SBQ prepared by the Ministry of National Education (Kertil et al., 2021). Research has demonstrated that students encounter challenges in addressing these inquiries due to the extensive nature and intricate nature of the SBQ (Kablan & Bozkuş, 2021; Karakeçe, 2021; Sarıoğlu et al., 2023; Uzun & Ağaç, 2023). Nonetheless, a number of studies have proposed the hypothesis that SBQ has the capacity to support students’ skill development (Aslan Efe & İz, 2023; Sarıoğlu et al., 2023). To illustrate this, research has been conducted that demonstrates the efficacy of questions based on skills in improving students’ abilities (see Uzun & Ağaç, 2023). Moreover, the SBQ prepared by the Ministry of National Education was found to demonstrate proficiency in factual knowledge, procedural knowledge, and conceptual knowledge (Sanca et al., 2021).

Research endeavours directed towards elucidating the structural intricacies of the SBQ have also been incorporated within the extant literature (Çataldere, 2022; Ergün, 2021; Ünsal & Kaba, 2022). A review of research on the perspectives of teachers from various disciplines regarding SBQs reveals the challenges and opportunities encountered in the implementation of these questions (Bayburtlu, 2021; Erden, 2020; Karakeçe, 2021; Kertil et al., 2021). As Rindermann (2007) demonstrate, international comparisons underscore the global significance of skills-based education.

In order to enhance the efficacy of SBQ, it is imperative to comprehend the manner in which science teachers perceive and conceptually construct these questions. Cognitive structure can be defined as a hypothetical structure that demonstrates the relationships between concepts in an individual’s long-term memory (Shavelson, 1974). The word association test (WAT) is a widely utilized method in the field of educational research for the purpose of revealing these relationships (see Bahar et al., 1999; Alaca et al., 2020; Balbağ, 2018). The WAT aims to reveal perceptions and misconceptions about concepts and topics (see Bahar et al., 1999). The present study aims to utilize WAT to examine the cognitive constructions employed by science teachers in relation to SBQ.

The present study has been designed with the intention of determining the cognitive structures and perceptions held by science teachers with regard to skill-based questions.

Research Aim and Research Questions

This study aims to investigate science teachers’ cognitive structures and perceptions of skill-based questions. The following research questions were sought to answer to achieve its current purpose.

- What is the frequency distribution of the concepts when examining science teachers’ perceptions and cognitive structures regarding SBQ?
- What is the correlation between the SBQ-related concepts that emerged from the word association test administered to science teachers?

## 2. Materials and Methods

In this study, a descriptive survey model was used to determine teachers’ cognitive structures for skill-based questions. The descriptive survey model is generally used by researchers to reveal characteristics such as opinions, ideas, attitudes, and beliefs of a larger group of people about a subject or subject (Frankel & Wallen, 2011). Descriptive survey research describes data related to variables in the study.

### 2.1. Construction of the Word Association Test

To construct the Skill-Based Question Word Association Test (SBQ-WAT), a single key phrase was selected to act as the stimulus: “skill-based question.” The rationale for selecting the SBQ concept to be included in the word association test is that it plays an important role in science lessons and assessments. The format of the SBQ-WAT required teachers to list up to five words they associated with the stimulus phrase within 30 s, followed by constructing a meaningful sentence using the phrase. They were then instructed to construct a meaningful sentence using this phrase (Bahar et al., 1999; Sikumbang et al., 2019; Yun, 2020). The stimulus phrase was written at the top of the response form and repeated five times down the side of the page to minimize the chaining effect, ensuring that each response remained tied to the stimulus rather than to previous responses.

### 2.2. Participants

During the 2021–2022 academic year, data was collected from 67 science teachers working in eastern Anatolia, Turkey. Snowball sampling was used to collect the data. Teachers were contacted in person and online. The snowball sampling method consists of including people who can provide the most information about the research subject in the sample (Patton, 2014). The SBQ-WAT was administered in person to some teachers and online to others. Data from five teachers were excluded due to repetitive responses. Teachers with unusable data were excluded from the evaluation because they wrote the same words in five lines and used the same answers for three words.

The participants in the study and their demographic information are provided in Table 1.

From Table 1, 54% of the evaluated teachers are female, and 39% are male. Most participating teachers are experienced, as shown by their seniority. The structure of science teachers suggests a significant difference between the number of individuals who have participated in skills-based activities and those who have not. Specifically, the number of individuals who have not participated in such activities is four times higher than those who have. Similarly, the number of individuals who have not prepared skill-based questions is three times higher than those who have.

### 2.3. Data Analysis

The statements pertaining to SBQ made by the teachers were collected in their verbatim form. The data presented herein was coded in accordance with teachers’ perceptions and concepts about SBQ. In an attempt to provide a comprehensive response, the following approach was taken: responses were related to specific characteristics of SBQ and were focused exclusively on statements related to the characteristics of multiple-choice questions.

The responses were then subjected to labelling according to SBQ’s three main themes and nine categories under these themes, for a total of 16 codes. Multiple-choice question characteristics were researched in the literature, and the characteristics that multiple-choice questions should possess were accepted as categories. The codes were distributed among the categories. The following themes are to be considered:

- Structure and Context: The following elements must be considered: question text and root, length, context of the questions and literacy.
- Validity and reabilityt: The following criteria are to be considered: options homogeneity, validity, difficulty, and usefulness.

The responses of each teacher were then subjected to coding in accordance with the characteristics they indicated.

- Should the response in question contain the relevant characteristic, the appropriate response would be ‘1’ (i.e., ‘present’).
- In the event that the response did not contain the relevant characteristic, the response should be designated as 0 (absent).
- In the event that the participant did not utilize any expression related to this characteristic, the field is left blank.

To illustrate this, consider the following example. If an instructor were to imply that the body text of SBQ is cognitive-based, then the ‘cognitive-based body text’ code would receive a value of 1, while all other body text codes would receive a value of 0. If the participant has not provided any statements with regard to this component, the relevant field is left blank.

Thematic and categorial frequency tables have been generated in order to summarize the responses provided by each participant. The following tables reveal which characteristics are most frequently associated with SBQ by teachers. In the analysis: Features with a frequency of 5 or higher are listed in this document. In contrast, those features exhibiting lower frequencies have been excluded. This approach has facilitated the identification of the concepts that are most commonly evoked by educators when encountering the term “SBQ”. Tables presents the aggregate number of distinct responses provided for each code, category, and theme. As Shavelson (1974) demonstrated, the counting of responses given to each code is a method of summarizing word association test data.

The Relatedness Coefficient, hereafter referred to as RC, was calculated in order to evaluate the strength of the relationships between concepts related to SBQ among teachers. The following sequence of events transpired:

- The coded data (i.e., 1, 0 or blank values) were analyzed using Kendall’s Tau, a statistical method used to measure the strength and direction of a relationship between two variables.
- The coefficient demonstrated the frequency with which disparate concepts manifested concomitantly within educators’ conceptualizations (indicating the potency of the correlative relationship).
- The RC values that were calculated formed the basis for understanding which concepts were evoked when SBQ was mentioned.

The creation of a network of relationships has been facilitated by the implementation of the RC values. The correlation coefficients have been ordered according to their magnitude, with the most significant coefficients assigned the highest rank. Subsequently, a systematic reduction has been applied at the 0.15 cut-off point, thereby establishing a network structure that progresses from concepts with strong relationships to those with weaker relationships. This network is employed to illustrate the conceptual connections that are more firmly entrenched within the cognitive frameworks of teachers pertaining to SBQ.

Whilst it is to be expected that each code will demonstrate a high negative correlation with other codes in the same category, given that they are alternatives to each other, they were not included in the calculations. This was due to the belief that it would complicate the interpretation of the data in this study. For instance, the utilization of a cognitive body text statement would signify an absence of experience. Consequently, while the correlation between these two codes would be negative and substantial, the calculation of the correlation coefficient was not undertaken.

This comprehensive analysis has unveiled a multifaceted perspective, offering insights into teachers’ perceptions and cognitive frameworks concerning SBQ. Descriptive analysis was instrumental in categorizing the responses obtained, while MRC and the relationship network were found to be significant tools for understanding the mental organization of these concepts. Consequently, a comprehensive dataset was collated pertaining to the function of SBQ within educational settings and the manner in which pedagogues interpreted these questions

## 3. Results

In word association tests, the number of different responses that individual users give for specific codes is an important, direct indicator of the degree to which a particular word is associated with a related concept. This is attributable to the notion that meaning can be delineated in proportion to the number and intricacy of connections an individual user is capable of forging with a particular word. The correlations that can be established between the statements used by educators with regard to SBQs are also directly related to this number. In summary, the plethora of valid statements employed signifies that the conceptualization under investigation is more intimately associated with the terminology employed. In the absence of connections, a word possesses a relatively limited degree of meaning; however, as connections are established, the word’s meaning becomes more complex and nuanced (Brooks & Brooks, 1999).

Participant responses were transferred to word processing programmes. Then, statements expressing judgments were extracted and listed. The reported judgments were coded as keywords. Then, it was decided under which headings these codes could be grouped, and these were transformed into categories. The categories were then separated into themes based on the qualities required of multiple-choice questions and are presented in Table 2.

Table 2 presents the various codes obtained from the statements employed for SBQ, along with their respective frequencies. The term ‘mental body text’ is frequently cited as a response to SBQ, and it is notable that this response is mentioned more often than other codes such as ‘culture-sensitive’, ‘requires interpretation’, and ‘heterogeneous option’. The prevalence of the keywords ‘cognitive-based question text and root’ and ‘requires interpretation’ may be attributable to the fact that students preparing for exams are abstracted from the real world during the exam preparation period and are required to focus solely on exam preparation. In preparation for the SBQ, students focus on solving a variety of questions to increase their familiarity with different question types and to become acquainted with questions that are likely to appear in the entrance exam for qualified high schools. This phenomenon may result in the subjects adopting a Kantian perspective, characterized by a prolonged engagement with cognitive thought. The fact that the SBQ-style questions that will appear in the Higher Education Entrance Exam (LGS), arguably the most significant first major examination in the Turkish education system, are far from achieving their intended effect is made even more apparent by the significant number of codes such as ‘heterogeneous choices,’ ‘low validity,’ ‘literacy,’ and ‘low usefulness.’ This assertion is corroborated by teachers’ descriptions of SBQ, which utilize terms such as ‘logic,’ ‘knowledge,’ ‘interpretation,’ ‘intelligence,’ and ‘reasoning.’ Conversely, the utilization of terminology such as ‘PISA and TIMSS-like,’ ‘new generation,’ and ‘21st century skills’ by certain educators signifies their conviction that these SBQs have been meticulously prepared. It has been observed that the other statements of those who prefer these terms are generally coded in a positive manner. The views expressed by these teachers may be attributable to their conviction that administering an examination analogous to international assessment and evaluation initiatives is congruent with the nation’s educational policy of ‘attaining the level of contemporary civilizations’. This conviction is further underpinned by their belief that the examination is designed to impart the competencies deemed essential for contemporary age.

The fundamental assumption of WAT is that the sequence in which responses are retrieved from long-term memory reflects, at least in part, the underlying structure of concepts within and between individuals (Fitzpatrick & Izura, 2011; Jonassen, 1987; Shavelson, 1974). In the context of word association tests, the degree of overlap in response hierarchies has been identified as a measure of the semantic proximity of the stimulus words (Deese, 1965; Nelson et al., 2004). A substantial corpus of research studies has demonstrated that semantic memory structure is characterized by a high degree of order (see; Reilly et al., 2022; Folstein & Dieciuc, 2019; Kutas & Federmeier, 2011). The process of recalling concepts that are distant from each other in the hierarchical structure is known to require greater temporal investment. Concepts that are more closely related to each other in the hierarchy require less time to recall and are located at the centre of the meaning of concepts or categories. This phenomenon, referred to as “semantic relationship” or “semantic distance effect,” reveals a fundamental generalization: the closer two concepts are semantically, the faster information related to these concepts is recalled. This may explain the importance of the type of word teachers respond to for each cue word. To determine the extent to which each teacher associated a particular cue with another concept, Kendall’s Tau was calculated as a correlation coefficient for each code. Table 3 shows the values of the RC.

Upon examination of the data presented in Table 3, which illustrates the inter-code correlation coefficients, it becomes evident that none of the respondents who perceived the SBQs as lengthy questions expressed positive sentiments regarding the literacy of the questions. The inverse of this relationship was also observed in those who perceived the questions to be challenging and culturally sensitive.

The negative perception of extended questions in terms of literacy indicates that care should be taken to ensure that questions are concise. The strong correlation between challenging questions and cultural sensitivity suggests that cultural context may play a significant role in the perception of difficulty. The evident literacy and validity of experience-based questions suggests that greater emphasis should be placed on such questions in education. The literacy of real-life contexts has been demonstrated to render questions more applicable and understandable. However, the negative impact of heterogeneous choices on literacy and validity indicates the need for attention to homogeneity in option design.

**Figure 1:**
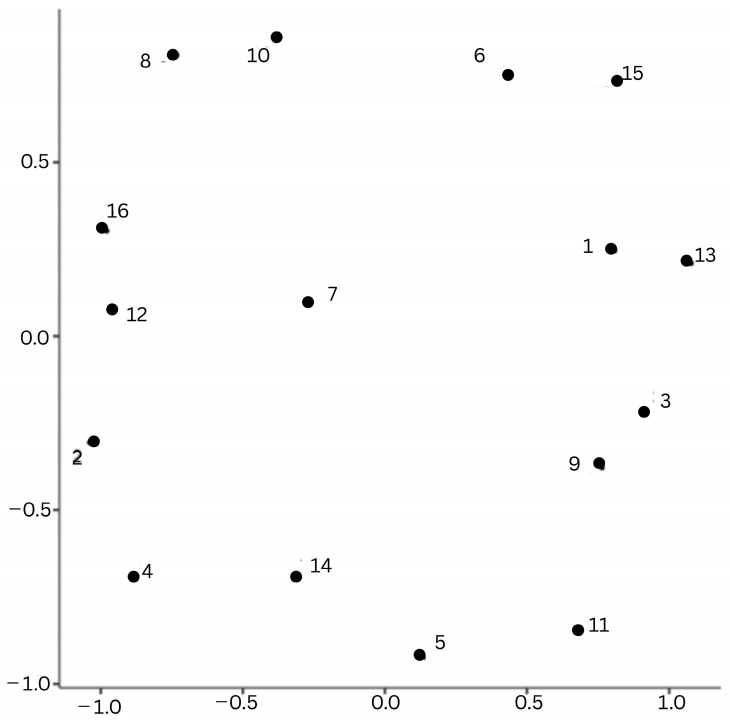
Correlation Map of Variable. Note: 1—Cognitive-based 2—Experience based 3—Long Question 4—Real Life Context 5—Interdisciplinary context 6—Requiring Comment 7—Culturally Context Consistency 8—Context no fitting the Culture 9—Literacy 10—Homogeneous Choise 11—Heterogeneous Choise 12—High validity 13—Low validity 14—Difficult question 15—Low usefulness 16—High usefulness.

The correlation map, which visualizes the relationship between data based on correlation coefficients, is shown in Figure 1.

Kendall’s Tau correlation coefficients between categories were presented as a correlation map. As suggested by Zhang et al. (2015), in this stage, the distances between points were first calculated based on the correlation coefficients between variables. The formula (√(2 − (1 − r))) was used for this purpose. Possible coordinates were determined by 16 points according to the distances. These coordinates were then visualized using a scatterplot graph. This figure illustrates the arrangement of 16 codes within a two-dimensional space, as determined by their correlation matrix. Variables that demonstrate a high degree of positive correlation are positioned in closer proximity to each other, while those exhibiting a high degree of negative correlation are positioned at a greater distance from each other. The correlation coefficients in the matrix were used to create the map.

This analysis suggests that certain codes are more likely to be connected to particular networks compared to other networks, indicating potential synergies and competitive dynamics among different codes. The finding that the phrases “cognitive-based question text and root” and “heterogeneous choice” are most frequently used together, followed by “low validity,” is indicative of the proximity of these three concepts. The recurrent and concurrent utilization of these expressions may signify a cognitive framework whereby the efficacy of SBQs is deemed inadequate due to their inherent cognitive-based characteristics and the heterogeneity of the available options. Conversely, the code designated as ‘culturally sensitive’—predominantly observed in phrases that demonstrate the appreciation of differences in SBQs and the promotion of critical thinking—was held in high regard by all those who assert that SBQs were ‘difficult questions’. This suggests a degree of perplexity among educators. The rationale behind this phenomenon can be traced back to the manner in which questions of a challenging nature are received. It has been observed that the acceptance of such questions can be interpreted as a catalyst for an augmented preparation period. Despite the cultural diversity that is held in high regard, there is a prevailing sentiment that the probability of individuals encountering such experiences is minimal.

**Figure 2:**
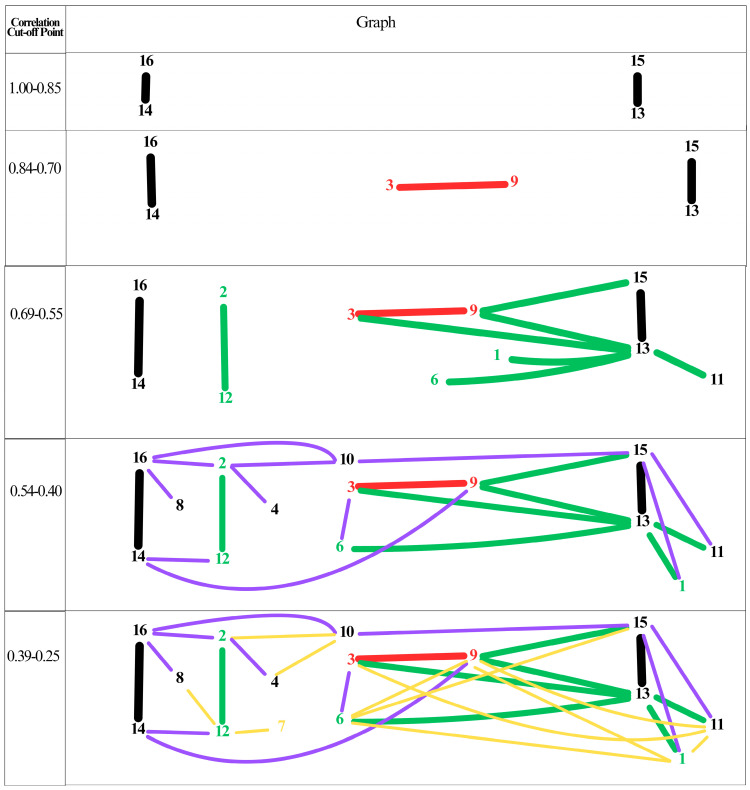
Teachers’ cognitive structure towards SBQ created using Kendall’s Tau. Note: 1—Cognitive-based 2—Experience-based 3—Long Question 4—Real Life Context 5—Interdisciplinary context 6—Requiring Comment 7—Culturally Context Consistency 8—Context no fitting the Culture 9—Literacy 10—Homogeneous Choice 11—Heterogeneous Choice 12—High validity 13—Low validity 14—Difficult question 15—Low usefulness 16—High usefulness.

In an effort to comprehend the cognitive framework that teachers employ in regard to SBQs, cut-off points were ascertained through the gradual diminution of correlation coefficients. The resulting graphical representation is presented in Figure 2.

The correlation coefficients presented in Figure 2 can also be utilized to assess the relationship between each science teacher and a specific stimulus. Figure 2 provides a visual representation of the relationships between the codes, from the most dominant to the least dominant, according to their preference by different users. The standard deviation value of 0.15 was determined as the correlation cutoff point for the 136 unique correlation coefficients calculated among the 16 codes. To align with the 15 features of the mean of 100 standard deviations in the interpretation of Stanford IQ tests, a score of 0.15 was chosen. This score represents 15% of the maximum score of 1 and maintains the standard of interpretation. A graph was then created by gradually reducing the values from the highest correlation coefficient to the lowest significant correlation coefficient.

As demonstrated in Figure 2, the relationship between the ‘culturally sensitive’ and ‘difficult question’ codes is the strongest, given that these two codes are utilized in a similar manner by all users. In addition to these codes, ‘low validity’ and ‘low usefulness’ exhibit a strong correlation, indicating a close relationship between these concept pairs. The establishment of three distinct pairs, in the absence of discernible relationships between them, signifies that the conceptualization of the phenomenon remains ambiguous.

**Figure 3:**
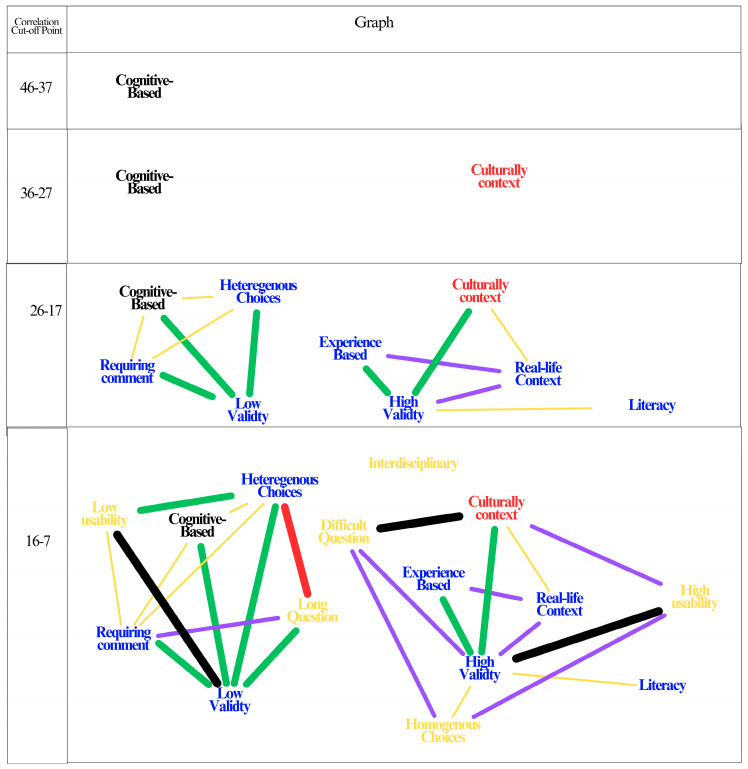
The interconnectedness between codes using word frequencies.

Participants were asked the words that came to mind when SBQ was mentioned, and Figure 3 was created based on the answers given.

The ‘map’ constructed utilizing the frequency of each code selection (see Figure 3) elucidates both the frequency and significance of the codes in the teachers’ conceptual framework. The phrase “cognitive-based main text” is the most frequently occurring phrase in the highest frequency range. The second most frequently selected code, ‘Cultural sensitivity’, is similarly positioned alone in the second range. It is evident that this situation will undergo a transformation in subsequent steps. However, it is noteworthy that one of the two most frequently selected codes conveys a positive view, while the other conveys a negative view. This situation suggests that teachers are still experiencing some confusion regarding SBQ. Upon reaching the third range, novel codes emerge, and concepts related to each other begin to appear. In this study, four distinct relationships, categorized into two overarching clusters, have been identified. In this stage, where the number of relationships between positive codes is higher, it is understood that SBQs are particularly low in validity due to their ‘cognitive-based main text,’ and that their validity is high due to their cultural sensitivity, experience-based nature, and real-life context. In the subsequent step, it is understood that minds are clustered in two different nodes as the number of relationships increases. The codes have been observed to form isolated clusters, gradually coalescing to create meaningful networks. While 15 of the 16 codes coalesce in the final frequency range, one code is so infrequent (only appearing five times) that it cannot be displayed in the network. This phenomenon may be interpreted as a tendency among some teachers to omit specific characteristics of the SBQ in their responses.

## 4. Discussion

In this study, the cognitive structures of science teachers in Turkey regarding science skill-based questions were attempted to be constructed using a word association test. The concepts and words provided by the teachers were converted into specific codes (cognitive-based, experience based, long question, real life context, interdisciplinary context, requiring comment, culturally context consistency, context not fitting the culture, literacy, homogeneous choice, heterogeneous choice, high validity, low validity, difficult question, low usefulness, high usefulness) and the correlations between them were examined. It was observed that the connections between concepts were weak in some areas and much stronger in others.

The number of different responses given to a word is a significant and direct indicator of an individual’s comprehension of the word (Gökmen Yekeler & Ulusoy, 2022; Flekko et al., 1973). This is because meaning can be defined as proportional to the number and complexity of connections an individual can make with the word. The findings of this study suggest the necessity for interventions aimed at mitigating teachers’ uncertainty regarding SBQ. One possible intervention is for the Ministry of National Education to provide teachers with periodic training to ensure they maintain the necessary skills for students. It is recommended that the significance of the codes be increased for teachers, thereby increasing the average number of responses for each code. In other words, the number of “valid responses” to a code may be proportional to the meaningfulness of the key concept. In the absence of connections, a word possesses a relatively limited degree of meaning; however, as connections are established, the word’s meaning becomes more complex and nuanced (Brooks & Brooks, 1999).

The codes generated based on the different responses to the trigger word “SBQ” include “cognitive based”, “cultural context consistency”, “literacy” and “heterogeneous choices” these are seen more frequently than others. The fact that teachers require higher-order thinking for SBQ and can relate it to literacy means that they are aware of the skills that need to be measured for students. A significant limitation of this study is that no research was conducted on the nature of the experiences revealed by the questions. The reason for this is that experiences need to be utilized to explain the reasons behind the answers in detail. However, this approach was not adopted due to the belief that teachers have the ability to abstract. It is assumed that this issue could be addressed in subsequent studies by asking teachers to provide the rationale for their abstractions.

Responses from educators using terms such as “PISA,” “TIMSS,” “new generation,” and “21st century skills” were mostly interpreted positively. However, statements such as “reading comprehension skills are very important,” “there are tables and graphs,” and “the questions are very comprehensive” reflected a conflicting view. Starting from sentences within the scope of WAT, teachers stated that, in science SBQs, students can answer comprehension questions and questions with tables and graphs regardless of the importance of science concepts. The teachers’ responses were so different and contradictory that they could not be linked to each other, which may indicate diversity of opinion. However, this situation also shows that a common language has not yet been established in teaching, which may affect the interpretation of the data.

The present study has sought to shed light on the relationships between the main ideas reported by teachers. A comparison of this general picture with the objectives of decision-makers and assessment units in relation to SBQs will facilitate understanding of the functionality of the system. However, it is imperative to recognize the necessity for the Ministry of National Education to provide a comprehensive explanation of the underlying principles and implications of these questions prior to their utilization. It is possible that teachers may have focused on something unintended by the SBQ and overlooked an important connection. As previously stated, this evaluation serves as an example representing the sample of science teachers sample reached through snowball sampling.

Regarding skill-based questions, teachers mentioned encountering such question types on the High School Entrance Exam (LGS), PISA, and TIMSS. Studies in this area include those on central exams (Aslan Efe & İz, 2023; Tuna & Kapucu, 2022) and those investigating education systems and PISA (Kupiainen & Hautamäk, 2014). In our study, science teachers stated that the LGS and PISA exams included skill-based questions and that they had a high-order cognitive structure based on Bloom’s taxonomy. Çakır (2019) supported this finding by examining and comparing LGS and PISA exam questions based on Bloom’s revised taxonomy. According to Bloom’s revised taxonomy, the science questions in the LGS and PISA exams were mostly from the conceptual knowledge step. In the cognitive process dimension, the questions were mostly from the analysis, evaluation, and creation steps, which are considered high-level cognitive processes.

Turkey recorded an eight-point increase in the 2022 PISA science literacy test compared to the previous test (MoNE, 2022). Clearly, there has been a significant improvement in science performance on the 2022 PISA test compared to the 2018 test. Including skills in the science curriculum and integrating them into high school entrance exams has contributed to students’ success in international exams. Therefore, Turkey’s reasonable increase in success on international exams may be related to the importance given to these skills in the science curriculum and their measurement in exams. Examining science results in future international exams (PISA and TIMSS) and conducting a longitudinal study by analyzing the country’s success graph in science education could provide clearer insights into the country’s progress. Studies have discussed how the PISA 2022 results show that not only school curricula but also students’ different cultural contexts and socioeconomic levels affect their performance (Bruce et al., 2025; Song & Tan, 2025).

For future studies, it may be advisable to use mixed methods or triangulation. Researchers could expand beyond the context of science teachers to conduct longitudinal or comparative studies among different populations of teachers. These studies could provide detailed demographic information about participants and analyze teachers’ classroom practices alongside conceptual associations.

After skill-based questions were introduced in Turkey, the increase in PISA and TIMSS scores should not be overlooked by other countries. Researchers can delve deeper by gathering opinions from parents, teachers, and students. It is important to conduct studies on skill-based questions created using digital technologies.

## 5. Conclusions

This study examined the perceptions and cognitive structures of Turkish science teachers regarding skill-based questions. The teachers were asked to construct sentences that would reveal their perceptions. The results showed that the WAT had similar sentence structures. Upon examining the results, it was found that the teachers indicated that students developed negative attitudes. The teachers stated knowledge alone was insufficient and the questions were long and difficult. Another finding derived from the perceptions of science teachers is the debate over whether they possess the necessary skills for skill-based questions. Teachers’ sentences for skill-based questions were found to include expressions indicating the presence of one or more skills. Teachers also focused on the positive aspects of skill-based questions, such as their cognitively based nature, requirement of 21st-century skills, interdisciplinary content, high utility, and need for interpretation. Given the critical role of these types of questions in assessing higher-order cognitive skills, such as critical thinking and problem-solving, in line with global standards, such as PISA and TIMSS, it is important to understand how educators interpret them in order to apply them effectively.

This study examined the perceptions and cognitive structures of Turkish science teachers regarding skill-based questions. Teachers were asked to construct sentences that would reveal their perceptions. The results showed that the WAT had similar sentence structures. Upon examining the results, it was found that the teachers indicated that students developed negative attitudes. The teachers stated that knowledge alone was insufficient and the questions were long and difficult. There was a strong correlation between “cognitive-based” and “experience-based,” as well as between “high validity” and “high usefulness.” Consistent relationships were found between “long question” and “cognitive-based,” “literacy” and “long question,” and “high validity” and “experience-based.” The negative perception of literacy associated with long questions suggests that questions should be short and concise. Conversely, experience-based questions were found to be clear, valid, and easy to understand. The frequent co-occurrence of the terms “cognitive-based main text,” “heterogeneous options,” and “low validity” suggest that teachers find SBQs less effective due to these characteristics. These findings clearly demonstrate the urgent need for better teacher training and clearer guidelines for effectively using SBQs. Teachers also tend to focus on the positive aspects of SBQs, such as being cognitive-based, possessing 21st-century skills, being interdisciplinary, having high usability, and requiring interpretation. Given the critical role of such questions in assessing higher-order cognitive skills, such as critical thinking and problem-solving, and in line with global standards, such as PISA and TIMSS, it is crucial to understand how educators interpret these questions to implement them effectively.

It is vital that the gap between policy and practice be bridged and that adequate support be provided to teachers. Teachers’ uncertainty about the basic principles and outcomes of SBQs should be reduced and the meaning of key concepts made clearer by the Ministry of National Education (MEB) providing comprehensive explanations of these prior to their use. A number of pragmatic recommendations have been proposed, including the formulation of questions that are more concise by concentrating on questions based on experience. It has been demonstrated that such questions have a higher degree of literacy and validity. In addition, meticulous attention should be paid to the homogeneity of option design in order to enhance literacy and validity. Upcoming studies might expand this investigation by requesting that teachers clarify the reasons for their abstractions, offering more comprehensive understandings of the experiences that underpin their answers.

## Figures and Tables

**Table 1:** Demographic Information for Participants.

Variables	Demographic Features	N	%
Gender	Female	36	54
	Male	26	39
Seniority	1–6 years	13	20
	7–12 years	18	27
	13–19 years	18	27
	20 years and above	13	20
Status of receiving training on skill-based question	Yes	13	20
	No	49	73
Status of skill-based question preparation for exams, books and etc.	Yes	17	25
	No	45	68

**Table 2:** Frequencies of SBQ-related themes, categories and codes.

Theme	Category	Code	Samples	Frequency
Structure and Context	Question Text and Root	1—Cognitive- based	Reading comprehension, Thinking skill, Reasoning, Analytical thinking	46
		2—Experience based	21st century experience, Life skills, Related the daily life	17
	Length	3—Long question	Long question, Long, Exaggerated	13
	Context	4—Real Life Context	Daily life, Next generation, transformation of knowledge into skills	22
		5—Interdisciplinary context	Relates, new synapse connections, multidisciplinary, Multiple thinking	10
		6—Requiring comment	Complex, Comment Knowing not enough	26
		7—Culturally context consistency	Everyday life adaptation, Cultural life skills	34
		8—Context no fitting the culture	Difficulty in implementing nationwide	5
	Literacy	9—Literacy	Science literacy Requires attention, Proficiency in one’s language	26
Validity and Reliability	Options Homogeneity	10—Homogeneous choices	Misleading, Resembles distractors	9
		11—Heterogeneous choices	Reading ability	26
	Validity	12—Highly validity	PISA similarity –TIMSS similarity, compatibility with the curriculum	22
		13—Low validity	Fewer science concepts, reading speed, just reading, just explain the table	21
	Difficulty	14—Difficult question	Difficulty, difficult question questions that students are afraid of, The nightmare of family	13
	Usefulness	15—Low usefulness	Students’ can’t solve Generally long question	11
		16—High usefulness	Educational questions leads to argumentation Enables an active classroom environment	9

**Table 3:** Kendall’s Tau Coefficients for code matches.

Cat.	Code	1	2	3	4	5	6	7	8	9	10	11	12	13	14	15	16
**A**	**1**	—															
	**2**	−1.0	—														
**B**	**3**	0.65	−0.65	—													
**C**	**4**	−0.43	0.43	−0.64	—												
	**5**	0.19	−0.19	0.18	−0.36	—											
	**6**	0.28	−0.28	0.46	−0.71	−0.4	—										
	**7**	−0.14	0.14	−0.15	NaN	NaN	NaN	—									
	**8**	−0.06	0.06	−0.77	0.14	−0.23	0.03	0.16	—								
**D**	**9**	0.37	−0.37	0.77	−0.52	0.2	0.35	−0.28	−0.67	—							
**E**	**10**	−0.32	0.32	−0.26	0.29	−0.1	−0.19	NaN	0.22	−0.39	—						
	**11**	0.32	−0.32	0.26	−0.29	0.1	0.19	NaN	−0.22	0.39	−1.0	—					
**F**	**12**	−0.65	0.65	−0.63	0.50	0.03	−0.51	0.30	0.34	−0.61	0.55	−0.55	—				
	**13**	0.62	−0.62	0.63	−0.57	0.01	0.56	−0.30	−0.32	0.57	−0.59	0.59	−0.96	—			
**G**	**14**	−0.15	0.15	NaN	0.21	0.1	−0.30	0.17	−0.22	0.40	0.08	−0.05	0.41	−0.41	—		
**H**	**15**	0.54	−0.54	NaN	−0.33	0.02	0.29	−0.25	−0.41	0.56	0.54	−0.54	−0.88	0.88	−0.29	—	
	**16**	−0.41	0.41	NaN	0.15	0.0	−0.15	−0.19	0.41	−0.56	0.46	−0.46	0.88	−0.88	0.22	−0.91	—

Cat. (Category): A—Question Text and Root B—Length C—Context D—Literacy E—Options F—Validity G—Difficulty H—Usefulness. Code: 1—Cognitive-based 2—Experience based 3—Long Question 4—Real Life Context 5—Interdisciplinary context 6—Requiring Comment 7—Culturally Context Consistency 8—Context no fitting the Culture 9—Literacy 10—Homogeneous Choise 11—Heterogeneous Choise 12—High validity 13—Low validity 14—Difficult question 15—Low usefulness 16—High usefulness.

## Data Availability

The raw data supporting the conclusions of this article will be made available by the authors on request.

## Abbreviations

The following abbreviations are used in this manuscript:

SBQ	Skill Based Question
WAT	Word Association Test
MoNE	Ministry of Education
